# Evolution of impulsivity levels in relation to early cannabis use in violent patients in the early phase of psychosis

**DOI:** 10.1017/S0033291721005316

**Published:** 2023-05

**Authors:** Elise Dan-Glauser, David Framorando, Alessandra Solida-Tozzi, Philippe Golay, M. Mehdi Gholam, Luis Alameda, Philippe Conus, Valerie Moulin

**Affiliations:** 1 Department of psychiatry, Institute of Forensic Psychiatry, Lausanne University Hospital, Lausanne, Switzerland; 2 Institute of Psychology, Lausanne University, Lausanne, Switzerland; 3 Department of Psychiatry, Service of General Psychiatry, Lausanne University Hospital, Lausanne, Switzerland; 4 Department of Psychiatry, Center for Psychiatric Epidemiology and Psychopathology, Lausanne University Hospital, Lausanne, Switzerland; 5 Department of Psychosis Studies, Institute of Psychiatry, Psychology & Neuroscience, King's College London, London, UK; 6 Instituto de Investigación Sanitaria de Sevilla, Hospital Universitario Virgen del Rocío, Department of Psychiatry, Universidad de Sevilla, Sevilla, Spain; 7 Service of General Psychiatry, Lausanne University Hospital, Lausanne, Switzerland

**Keywords:** Cannabis use, early cannabis use, early-phase psychosis, impulsivity, violent behavior

## Abstract

**Background:**

Prevention of violent behaviors (VB) in the early phase of psychosis (EPP) is a real challenge. Impulsivity was shown to be strongly related to VB, and different evolutions of impulsivity were noticed along treatments. One possible variable involved in the relationship between VB and the evolution of impulsivity is cannabis use (CU). The high prevalence of CU in EPP and its relationship with VB led us to investigate: 1/the impact of CU and 2/the impact of early CU on the evolution of impulsivity levels during a 3-year program, in violent and non-violent EPP patients.

**Methods:**

178 non-violent and 62 violent patients (VPs) were followed-up over a 3 year period. Age of onset of CU was assessed at program entry and impulsivity was assessed seven times during the program. The evolution of impulsivity level during the program, as a function of the violent and non-violent groups of patients and CU precocity were analyzed with linear mixed-effects models.

**Results:**

Over the treatment period, impulsivity level did not evolve as a function of the interaction between group and CU (coef. = 0.02, *p* = 0.425). However, when including precocity of CU, impulsivity was shown to increase significantly only in VPs who start consuming before 15 years of age (coef. = 0.06, *p* = 0.008).

**Conclusion:**

The precocity of CU in VPs seems to be a key variable of the negative evolution of impulsivity during follow-up and should be closely monitored in EPP patients entering care since they have a higher risk of showing VB.

## Introduction

Compared to the general population, people suffering from psychosis (Arseneault, Moffitt, Caspi, Taylor, & Silva, 2000; Hodgins, 1992; Hodgins, Piatosa, & Schiffer, 2014; Tiihonen, Isohanni, Räsänen, Koiranen, & Moring, 1997; Wallace, Mullen, & Burgess, 2004), and especially those in the early phase of psychosis (EPP) (Chang et al., 2015; Large & Nielssen, 2011; Large, Dall, & Nielssen, 2014; Spidel, Lecomte, Greaves, Sahlstrom, & Yuille, 2010; Winsper et al., 2013b), exhibit a high prevalence of violent behaviors (VB) against others. The prevention of these behaviors in treatment programs for early psychotic disorders is of major importance since studies have shown that a significant proportion of patients displays assaults during these programs (Coid et al., 2013; Large & Nielssen, 2011; Moulin et al., 2018b; Winsper et al., 2013a). A decrease in VB would lead to less patient stigmatization and improve their social integration. In this respect, mental health services have a key role to play in the assessment and prevention of VB.

Unfortunately, the development of preventive strategies is still hampered by a lack of understanding of the mechanisms underlying these VB. Recent work has shown that within the heterogeneous group of violent patients (VPs) identified in the literature, impulsivity may play a central role in the occurrence of VB (Harris et al., 2010; Moulin et al., 2018b). Specifically, impulsivity [defined as ‘the tendency to react in an excessive, non-elaborated way, and to ignore the consequences of actions (Moeller, Barratt, Dougherty, Schmitz, & Swann, 2001)*’*] *was* shown to trigger deleterious effects, worsening clinical outcomes and complicating patient treatment and management in psychosis (Abdel-Baki, Turgeon, Chalfoun, & Nguyen, 2013; Hoptman, 2015; Kaladjian, Jeanningros, Azorin, Anton, & Mazzola-Pomietto, 2011; Volavka & Citrome, 2008). Moreover, prospective early psychosis cohort studies have revealed that impulsivity was associated with serious aggression (Harris et al., 2010; Moulin et al., 2018b) and a meta-analysis and a meta-regression analysis of psychopathological risk factors of VB in psychosis (EPP or chronic patients) have shown that high impulsivity was an important variable when considering VB (Rund, 2018; Witt, Van Dorn, & Fazel, 2013). Even more relevant, a recent analysis of impulsivity levels, over a 3-year treatment period, in two groups of patients (violent and non-violent) has shown a differential evolution of impulsivity according to the groups (Moulin et al., 2018b). This point deserves further investigation, as the varying evolutions of impulsivity levels in some patients could be indicative of the presence of particular risk factors for the recurrence of VB during treatment.

Interestingly, various authors suggest that the relationship between impulsivity and VB may be mediated by substance use disorder (Bjørkly, 2013; Ouzir, 2013; Ramírez & Andreu, 2006; Volavka & Citrome, 2008). The literature also suggests that these two variables are related to each other, and that they feed themselves. Particularly, it was suggested that repeated cannabis use (CU) may increase impulsivity (Chanut, 2013; Gonzalez et al., 2012; Le Moal & Koob, 2007; Tiihonen & Swartz, 2000) and that, conversely, impulsivity may play a key role in the development and maintenance of drug dependence, including cannabis (Allen, Moeller, Rhoades, & Cherek, 1998; Chanut, 2013; Green et al., 2004; Gut-Fayand et al., 2001; Ouzir, 2013; Swartz et al., 1998). Further, CU has been reported to be highly prevalent among young people with psychosis (Koskinen, Löhönen, Koponen, Isohanni, & Miettunen, 2009; Schoeler et al., 2016a) and high rates of CU are highlighted in EPP (29–38%) (Harris et al., 2010; Hodgins et al., 2011; Moulin et al., 2018a; Rolin et al., 2018), particularly in VPs (range between 44% and 64% (Harris et al., 2010; Hodgins et al., 2011; Moulin et al., 2018a; Rolin et al., 2018)). Besides, in EPP, CU has been shown to be a risk factor for serious violence (Dellazizzo et al., 2019; Moulin et al., 2018a; Rolin et al., 2018), even at a low dose (Harris et al., 2010). It was also suggested that VB could be potentiated by the precocity of CU (Rioux et al., 2018). Research has also shown that precocity of use increased the risk of substance used disorders in later life, but, more importantly, that the precocity of use before the age of 15, was also related to delinquency, may predispose to VB (Rioux et al., 2018; Schoeler et al., 2016b) and was a risk factor of VB in EPP (Moulin et al., 2020).

Considering that: 1/ impulsivity is an important factor in VB and could be the target of treatment programs, particularly as a decrease in impulsivity could lead to a decrease in VB; that 2/ we have observed different evolutions of impulsivity over the treatment period, showing that impulsivity did not decrease significantly in VPs (Moulin et al., 2018b), that 3/ CU could play an important role within the relationship between impulsivity and VB; and that 4/ precocity of CU may be a risk factor in the occurrence of VB, we decided to explore the links between the precocity of CU and the evolution of impulsivity levels in EPP patients. In order to characterize different patient profiles and identify the interaction between CU and violence in the evolution of impulsivity, we decided to observe separately VP and NVP during a three-year intervention program. Our hypothesis was as follows: the precocity of CU should significantly and differentially impact the evolution of impulsivity in VP and NVP groups during the program.

## Methods

### Participants and procedure

The patients included in this study stem from a cohort of 265 patients treated in a specialized early psychosis program, at the Department of Psychiatry at the University Hospital in Lausanne (Baumann et al., 2013; Conus & Bonsack, 2004). The entry criteria in the program were as follows: (i) to be aged between 18 and 35 years old; (ii) to reside in the catchment area; and (iii) to meet the threshold criteria for psychosis, as defined by the ‘Psychosis threshold’ subscale of the Comprehensive Assessment of At Risk Mental States Scale (Yung et al., 2005). The exclusion criteria were (i) antipsychotic medication taken for more than a total of 6 months, (ii) psychosis related to intoxication or organic brain disease, or (iii) intelligence quotient below 70.

All the patients were followed up prospectively over 36 months. Of the 265 patients, 15 were excluded from the analyses because the violence was not directed against people (did not involve harm to people, e.g. theft, drug trafficking), and 10 further patients were excluded because VBs were committed only before the entry to the program, leaving 240 patients included in the present study. Patients were classified as (a) ‘violent patients’ (VPs), having committed physical aggression against people at least once; or (b) ‘non-violent patients’ (NVPs), having committed no violent action (see the *Assessment of violent behavior* section below for further details on the definition of VB).

Case managers (CMs) completed a specially designed questionnaire for all patients. This questionnaire assessed demographic characteristics, medical history, history of VB, penal status, past treatment in forensic psychiatry, exposure to life events, as well as symptoms and functioning. The questionnaire has been filled with the information gathered from patients and their family over the first weeks of treatment and was updated during follow-up if new information emerged.

Follow-up assessments were conducted after 2, 6, 12, 18, 24, 30 and 36 months of treatment. They explored various aspects of treatment and co-morbidities, as well as the evolution of psychopathology and functional level. They were conducted by psychologists trained in the assessment of psychopathology and by CMs for descriptive measures. Psychopathology was assessed with The Positive and Negative Syndrome Scale (PANSS) (Kay, Flszbein, & Opfer, 1987) with good inter-rater reliability between psychologists.

The local Research and Ethics Committee granted access to the clinical data for research purposes (CER-VD; protocol #2020-00272).

### Measures

#### Diagnostic assessment

The diagnosis was the result of an expert consensus (between a psychiatrist and a psychologist) and was based on (1) the diagnosis reported by treating psychiatrists in all medical documents and at the end of any hospitalization, and (2) the longitudinal assessment by clinical CMs (Alameda et al., 2015). In this study, the main diagnosis according to the DSM-IV (Association, 1994) was taken into account for each patient. All the diagnoses were then subdivided into five classes.

#### Assessment of violent behavior

VB was defined as ‘*serious violence*’ (Large & Nielssen, 2011), which includes physical injury, sexual assault, and any use of a weapon against another person. Episodes of VB were identified: (1) by CMs, based on a validated questionnaire (Winsper et al., 2013a) allowing the recording of any violent offense and behavior during the clinical interactions occurring between them and the patients over the entire 36 month treatment period (averaging 100 contacts per patient); (2) by additional information provided by parents, significant others, and the forensic psychiatric services; and (3) based on the Staff Observation Aggression Scale [SOAS-R scale (Nijman et al., 1999)], which lists all critical events related to a VB during hospitalizations (VB during treatment).

#### Assessment of impulsivity

Impulsivity was assessed by combining the scores of 2 PANSS items (‘*poor impulse control*’ and ‘*difficulty in delaying gratification*’, on a seven-point scale), which corresponds to the definition of impulsivity proposed above (Moeller et al., 2001). Impulsivity was evaluated at program entry and at each assessment time point.

#### Assessment of substance use and its precocity

The age of onset of substance use was evaluated using the DSM-IV substance use history (Association, 1994). According to the literature, we have classified as ‘early cannabis users’, patients who have started CU before or until the age of 15; and as ‘late cannabis users’, patients who started after the age of 15. Current substance use was assessed by CMs upon patient entry to the program, based on the Case Manager Rating Scale (CMRS) (Drake et al., 1990). The CMRS rates the intensity of substance use (alcohol and CU) on a scale from 1 to 5 (1 being the absence of substance use and 5 being very severe substance use). The severity of substance use was assessed by its duration, and its effects on personal, psychological, professional and social functioning. The assessment was based on self-reports, interviews, behavioral observations, and collateral reports (family, group home, day center, community, etc.) (Drake et al., 1990). Consumption was coded by CMs at the time of entry to the program and at each time point of the follow-up.

### Statistical analysis

Differences between groups in terms of demographic data were assessed with chi-square and Student *t* tests. To answer our research question we first assessed to what extent impulsivity level at the entry into the program was different between VP and NVP, whether these patients were users or not. To do this, we conducted a two-way ANCOVA with two factors, Patient Group (VP *v.* NVP) and use (users *v.* Non- users), while controlling for the level of alcohol consumption at the entry into the program. We also further examined with the same method what were the differences in the level of impulsivity between early and late consumers, in relationship to the group (VP *v.* NVP).

To assess the evolution of impulsivity during the program and analyze the relationship between Group and CU, we compared the VP and NVP and the presence or absence of CU on impulsivity with a linear mixed-effects model, which included a random intercept for each individual. Time was included as a covariate to assess the evolution, with observations at 2, 6, 12, 18, 24, 30 and 36 months from the entry into the program. We used restricted maximum likelihood estimation (Pinheiro, Bates, DebRoy, Sarkar, & Team, 2007) to assess the effect CU on impulsivity levels of patients in the two groups of patients (VP *v.* NVP). Due to the longitudinal nature of the study, all models were adjusted for the inter-correlation among observations corresponding to the same individuals by using a random intercept for individual level. The model fit seemed to be satisfactory and using the penalized quasi-likelihood method we observed similar results. The same analysis was conducted to assess the effect of early CU on impulsivity. For the later analysis, only cannabis users have been taken into account, observing the evolution of impulsivity across time as a function of two factors: the Patient Group (VP *v.* NVP) and the Precocity of Use [(Before and until 15 years of age = *Early v.* After 15 years of age = *Late* (without age limit)]. In order to control for potential confounding factor, the level of alcohol use at baseline was adjusted in all models.

## Results

### Characteristics of the violent and non-violent groups

Comparing VPs and NVPs showed that VPs were more likely to be men (*p* = 0.001), to have a lower level of education (*p* = 0.003) and no professional activity (*p* = 0.037); VPs were younger (mean age 22.7 years) than NVP (mean age 24.3 years) at the time of admission to the program (*p* = 0.015). The distribution of marital status was equivalent in both groups (*p* = 0.998).

In our sample [as we have shown in a previous study (Moulin et al., 2020)], the number of patients using cannabis or alcohol at program entry was higher in the VP group (72.41% for alcohol, 63.79% for cannabis) than in the NVP group (69.05% for alcohol and 34.14% for cannabis). Moreover, in the VP group, the average age of the onset of CU was 15.29 years (*v.* 15.93 years in the NVP group, *p* = 0.005), and the average age of the onset of alcohol use was 15.12 years (*v.* 16.97 years in the NVP group, *p* = 0.016). Details of the sample are shown in Table 1.

**Table 1.** Descriptive characteristics of the study sample according to violent and non-violent patients

Descriptive characteristics	Total sample	*Violent and non-violent groups*		
	*N* = 240	violent patients (*N* = 62)	Non-violent patients (*N* = 178)	*p* value
Gender (male), %(*n*)	67.5 (162)	87 (54)	60.7 (108)	**0.001**
Age at the entry in the program, M(s.d.)	23.91 (0.31)	22.68 (0.56)	24.34 (0.37)	**0.0154**
Civil status : single, %(*n*)	85.17 (201)	85.25 (52)	85.14 (149)	
Civil status: married, %(*n*)	8.47 (20)	8.2 (5)	8.57 (15)	
Civil status: divorced, %(*n*)	2.97 (7)	3.28 (2)	2.86 (5)	0.9981
Number of years at school, M(s.d.)	9.73 (0.19)	8.79 (0.34)	10.04 (0.22)	**0.0030**
Student, %(*n*)	20 (48)	11.29 (7)	23.03 (41)	
Professional activity, %(*n*)	14.17 (34)	9.68 (6)	15.73 (28)	
No professional activity, %(*n*)	65.83 (158)	79.03 (49)	61.24 (109)	**0.0374**
Previous violence, %(*n*)	10.19 (21)	35.71 (20)	0.67 (1)	**0**
School maladjustement, %(*n*)	31.67 (76)	41.94 (26)	28.09 (50)	0.0629
Duration of untreated psychosis, M(s.d.)	1.3 (0.14)	1.04 (0.22)	1.39 (0.18)	0.2166
Diagnosis				
Schizophrenia, %(*n*)	58.33 (140)	56.74 (101)	62.9 (39)	
Schizophreniform/brief, %(*n*)	9.58 (23)	11.24 (20)	4.84 (3)	
Schizoaffective disorder, %(*n*)	9.58 (23)	7.87 (14)	14.52 (9)	
Major depression with psychotic features, %(*n*)	4.58 (11)	5.06 (9)	3.23 (2)	
Bipolar disorder, %(*n*)	6.67 (16)	6.74 (12)	6.45 (4)	
Others, %(*n*)	11.25 (27)	12.36 (22)	8.06 (5)	0.3600
Substance use at program entry				
Alcohol, %(*n*)	69.91 (158)	72.41 (42)	69.05 (116)	0.7521
Cannabis, %(*n*)	43.3 (97)	63.79 (37)	36.14 (60)	**<0.0001**
Age of onset of substance use				
Alcohol, M(s.d.)	15.73 (0.17)	15.12 (0.26)	15.93 (0.21)	**0.0162**
Cannabis, M(s.d.)	16.47 (0.29)	15.29 (0.45)	16.97 (0.35)	**0.0052**

The *P* values in bold are significant.

Regarding the level of use, VPs had a higher level of cannabis consumption at baseline (average = 2.24), as compared to NVPs (average = 1.55).

### Impulsivity level at program entry and as function of the group and the precocity of CU

As indicated in another publication regarding this cohort (Moulin et al., 2018b), VPs also showed an impulsivity level at baseline that was significantly higher (3.48) than that observed in non-violent patients (average = 2.64, see Table 2).

**Table 2.** Evolution of substance use and impulsivity

Variable		Total sample (*N* = 240)	Non-violent patients (*N* = 178)	Violent patients (*N* = 62)
Level of alcohol use	Baseline [mean/5 (s.e.m.)]	1.81 (0.05)	1.78 (0.06)	1.88 (0.11)
	At 2 months	1.60 (0.05)	1.55 (0.06)	1.74 (0.11)
	At 6 months	1.66 (0.05)	1.61 (0.05)	1.79 (0.13)
	At 12 months	1.65 (0.05)	1.59 (0.05)	1.79 (0.10)
	At 18 months	1.62 (0.04)	1.61 (0.05)	1.64 (0.09)
	At 24 months	1.72 (0.05)	1.67 (0.06)	1.89 (0.13)
	At 30 months	1.69 (0.05)	1.71 (0.05)	1.62 (0.12)
	At 36 months	1.64 (0.05)	1.6 (0.05)	1.76 (0.12)
Level of cannabis use	Baseline [mean/5 (s.e.m.)]	1.73 (0.07)	1.55 (0.07)	2.24 (0.15)
	At 2 months	1.62 (0.07)	1.49 (0.07)	1.97 (0.14)
	At 6 months	1.49 (0.07)	1.37 (0.06)	1.81 (0.12)
	At 12 months	1.52 (0.06)	1.37 (0.06)	1.95 (0.14)
	At 18 months	1.42 (0.05)	1.31 (0.05)	1.74 (0.11)
	At 24 months	1.54 (0.07)	1.40 (0.07)	1.93 (0.15)
	At 30 months	1.46 (0.06)	1.35 (0.06)	1.79 (0.16)
	At 36 months	1.42 (0.06)	1.24 (0.05)	1.93 (0.17)
Level of impulsivity	Baseline [sum (s.e.m.)]	2.83 (0.10)	2.64 (0.10)	3.48 (0.25)
	At 2 months	2.79 (0.13)	2.57 (0.13)	3.50 (0.30)
	At 6 months	2.46 (0.09)	2.38 (0.09)	2.84 (0.24)
	At 12 months	2.57 (0.11)	2.35 (0.09)	3.65 (0.39)
	At 18 months	2.63 (0.12)	2.39 (0.09)	3.71 (0.44)
	At 24 months	2.70 (0.12)	2.51 (0.10)	3.60 (0.46)
	At 30 months	2.74 (0.14)	2.44 (0.11)	3.95 (0.52)
	At 36 months	2.47 (0.11)	2.29 (0.08)	3.17 (0.44)

There was also a significant interaction between current CU (user *v.* non-user) and the group belonging (VPs *v.* NVPs), *F*_(1183)_ = 12.88, *p* = 0.006. Contrasts for this effect have shown that VPs who are current cannabis users have significantly higher levels of impulsivity (average level: 4.17) than any of the other group, i.e., the NVPs and VP that are not users (2.67 on average, *p* = 0.014 by Bonferroni posthoc tests).

There was no significant difference in impulsivity at baseline between early and late cannabis users *F*_(1,25)_ = 0.64, *p* = 0.430, and, there was no significant interaction between early use and the patient groups, *F*_(1,25)_ = 0.01, *p* = 0.924.

### Evolution of Impulsivity during treatment as function of the group and CU at baseline

Linear Mixed Effect model representing the evolution of Impulsivity level across the 3 years of treatment as a function of the group (VPs *v.* NVPs) and use (no use *v.* use) have shown no significant interaction between group, use and time in the Impulsivity level [coef = 0.02, 95%CI (−0.02 to 0.05], *p* *=* 0.425) (Table 3).

**Table 3.** Evolution of impulsivity over treatment as a function of the groups, cannabis use, and precocity of cannabis use

	Model 1			Model 2		
*Predictors*	*Estimates*	*CI*	*p*	*Estimates*	*CI*	*p*
(Intercept)	2.60	2.33–2.86	**<0.001**	2.84	2.32–3.36	**<0.001**
Violent	0.29	−0.22 to 0.80	0.256	0.63	−0.05 to 1.32	0.071
Cannabis use	−0.07	−0.64 to 0.51	0.811			
Time	−0.00	−0.01 to 0.00	0.342	0.00	−0.02 to 0.02	0.882
Alcohol use	−0.11	−0.37 to 0.15	0.413	−0.14	−0.40 to 0.12	0.297
Violent × Cannabis use.	0.79	−0.15 to 1.72	0.101			
Violent × Time	0.01	−0.01 to 0.03	0.181	0.04	0.01–0.07	**0.006**
Cannabis use. × Time	0.01	−0.02 to 0.03	0.540			
Violent × cannabis use × Time	0.02	−0.02 to 0.05	0.425			
No can. cons.				−0.33	−0.87 to 0.21	0.226
Age of can. cons.> 15				−0.25	−0.80 to 0.31	0.376
Violent × No can. cons.				−0.47	−1.44 to 0.50	0.340
Violent × Age of can. cons.> 15				0.46	−0.63 to 1.55	0.407
No can. cons.* Time				−0.00	−0.03 to 0.02	0.792
Age of can. cons.> 15 × Time				−0.00	−0.03 to 0.02	0.761
Violent × No can. cons. × Time				−0.03	−0.07 to 0.01	0.151
Violent × *Age of* can. cons. *> 15* × Time				−0.06	−0.10 to −0.02	**0.008**

*Note:* Model 1 includes evaluation of impulsivity along the 3 years of treatment (Time) as a function of the group (VP v. NVP) and cannabis use (Users v. Non Users at the time of entry into the program). Model 2 focuses on the Early v. Late consumers present in the VP or NVP groups to predict the evolution of impulsivity in Time.

The *P* values in bold are significant.

### Evolution of Impulsivity during treatment as function of the group and the precocity of CU

**Fig. 1.**
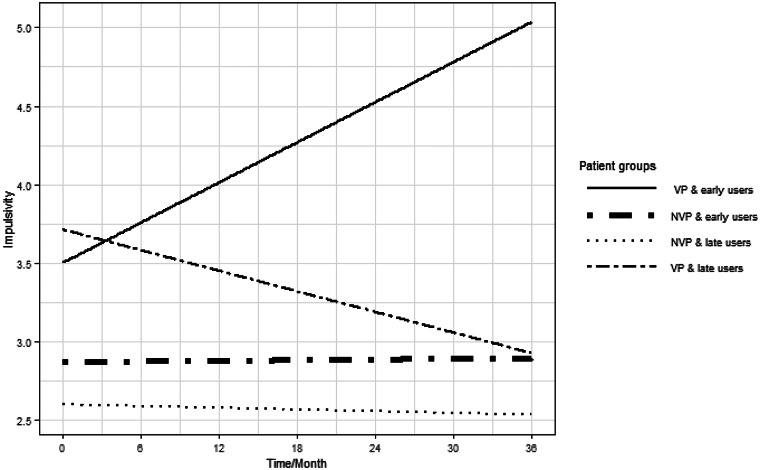
Evolution of Impulsivity during the 36 months of treatment as a function of the group and the precocity of cannabis use (Table-model 2).

Linear Mixed Effect model analyzing the evolution of Impulsivity level across the treatment as a function of the group (VPs *v.* NVPs) and precocity of use (before 15 years *v.* after) have shown that precocity of use in VP was linked to the level of impulsivity across time [coef = 0.06, 95%CI (−0.01 to −0.12) *p* *=* 0.008]. Based on the final model, NVPs and VPs with late CU behave similarly, with no or slight decrease (not significant) in impulsivity levels across the treatment. The VP group with early CU (before or at 15 years of age), displayed a significant increase in impulsivity level during the follow-up, ranging from 3.8 at 2 months to an average of 6 at 36 months (see Table 3 and Fig. 1).

In order to verify that the increase in impulsivity levels was not linked to a difference in CU between groups, we have compared the consumption trajectories of the groups over the time of the follow-up. There were no significant differences between the trajectories (early *v.* late and VPs *v.* NVPs): *F*_(6, 114)_ = 1.29, *p* *=* 0.270.

## Discussion

The purpose of this study was to analyze the relationship between CU, its precocity, and the variation of impulsivity levels during the treatment program, in violent and non-violent groups of patients. Our results showed that: (1) VPs who are cannabis users at program entry had significantly higher levels of impulsivity than the other groups studied, and that (2) there was a significant increase of impulsivity in the VP group whose patients are early cannabis users, whereas for the three other groups (i.e. the VPs who start CU after 15 years of age, the NVPs early user, i.e. before 15 years of age, and the NVPs who do not use cannabis), the impulsivity levels remained stable during the program**.** These results were significant even when data were controlled for the effect of alcohol use.

Various hypotheses could contribute to explain the involvement of early CU in the increase of impulsivity level during the treatment period. In the general population, impulsivity has already been associated to substance use, both in its early onset (Rioux et al., 2018) and in its maintenance (Vergés, Littlefield, Arriaza, & Alvarado, 2019). A recent review (Alford, O'Rourke, Doyle, & Todd, 2020) on impulsivity in the forensic population has shown for example, that the most robust factor associated with impulsivity was substance use, and it has been suggested that impulsivity could be simultaneously a determinant and a consequence of substance use (De Wit, 2009). In psychosis, it is unclear whether it is the pre-existing impulsivity trait that drives the consumption or whether it is the latter that increases impulsivity (Dervaux et al., 2010; Liraud & Verdoux, 2000; Ouzir, 2013). Various authors have shown that cannabis-user patients were more impulsive (more lack of control and difficulties in planning (Abdel-Baki et al., 2013; Dervaux et al., 2001; Dervaux et al., 2010)) than those who did not use. It was also suggested that CU may increase impulsivity (Chanut, 2013; Le Moal & Koob, 2007; Tiihonen & Swartz, 2000). Thus, the increase in impulsivity observed over the course of treatment could be linked to the self-feed between impulsivity and early CU.

A further hypothesis would be that early CU could have a stronger impact on the brain structures involved in impulsivity, as compared to later CU. Neurobiological research has found that CU during adolescence, which is a critical period for brain maturation (Paus et al., 1999), induces structural changes (Battistella et al., 2014; Churchwell, Lopez-Larson, & Yurgelun-Todd, 2010; Lopez-Larson et al., 2011; Wilson et al., 2000; Zalesky et al., 2012). These changes could particularly apply to brain structures involved in action control (Churchwell et al., 2010; Gruber & Yurgelun-Todd, 2005; Gruber, Dahlgren, Sagar, Gönenç, & Lukas, 2014; Gruber, Silveri, Dahlgren, & Yurgelun-Todd, 2011). Specifically, Churchwell et al. (Churchwell et al. 2010) reported that CU onset is related to the cortical volume of the orbitofrontal cortex, which is known to play a major role in impulsivity (Matsuo et al., 2009). In addition, Gruber (Gruber et al., 2011, 2014; Gruber & Yurgelun-Todd, 2005) has found a link between early-onset CU and differential brain activity during an inhibition task, as well as a stronger association between white matter integrity in the frontal areas and impulsivity scores. This result was found in early cannabis users (before the age of 16), compared to those exposed later. It is therefore likely that patients with early CU would be more prone to brain alterations in areas that are essential for action control, thus contributing to increased impulsivity levels. One last neurobiological hypothesis would be a combined effect of early CU on brain structure involved in impulsivity and progressive brain changes associated with the psychosis condition, both of which may impact impulsivity level (Hodgins et al., 2014).

However, these first explanations do not take into account that these results were not observed in patients who were early cannabis users and non-violent. Indeed, our results showed a specific increase of impulsivity in the violent-patients group who are early cannabis users, suggesting some specific vulnerability in this subgroup. So, examining the features of the living environment and the developmental trajectories of the violent-patients early-user could lead to a better understanding of what contributes to increased impulsivity in this group. In the general population, various studies suggest the effects of social environment, particularly the association with deviant peers, to explain early CU and its links with impulsivity and delinquency. For example, Rioux et al. (Rioux et al. 2018), have shown that impulsivity, involvement with deviant peers, and delinquency at age 12 predicted early onset of CU between 13 and 17 years of age, and this early-onset predicted future violence. Hence, delinquency, impulsivity and CU seem to be strongly associated, and studies suggest a link between pre-existing impulsivity, CU, and delinquency that may be self-feeding.

In psychosis, research has shown a particular profile of VP, with a complex developmental trajectory, characterized by the presence of early conduct disorders associated with antisocial behavior, including CU, and, this, before the age of 15 (Hodgins et al., 2014; Swanson et al., 2008; Volavka & Citrome, 2011). Early CU would be an integral part of these young's lives engaging in antisocial behavior (Dugré, Dellazizzo, Giguère, Potvin, & Dumais, 2017). In this patient profile, antisocial personality disorder was considered as an important condition involved in violence, which precedes and/or develops in parallel with psychotic disorders. The impulsivity observed in our study could be a manifestation of an antisocial personality disorder (Swanson et al., 2008; Volavka & Citrome, 2011) that develops early and/or a manifestation of an impulsivity trait that may be present early. This impulsivity trait could feed or be fed by early CU in this VPs group.

Since the increase of impulsivity level is specifically observed during the treatment period, it could also result from the effect of antipsychotic treatments, in VPs who are early cannabis users, in connection with the presence of a pre-existing impulsivity trait. However, while some studies have indeed shown that the choice of antipsychotic treatment may be important in the modulation of impulsivity and violence (Abdel-Baki et al., 2013; Hoptman, 2015; Nanda et al., 2016; Volavka & Citrome, 2008) in patients, we did not find any work specifically on the assessment of the evolution of impulsivity over time in VPs, and moreover showing a treatment effect that would increase impulsivity over time. In addition, we have verified that there was no relationship between positive symptoms and impulsivity levels, nor between treatment adherence and these levels.

In a more general way, it is increasingly recognized that mental health services specialized in the early treatment of psychotic disorders have a key role to play in the assessment and prevention of VB (Hodgins et al., 2009; Mullen, 2000). However, it seems that these specialized services are not yet adjusting their interventions, particularly regarding specific assessments and treatments for VP or for patients with delinquent history (Hodgins, 2017; Hodgins et al., 2009). Previous recommendations included (a) the use of valid VB risk assessment tools (Hodgins, 2017), which have shown their effectiveness in preventing VB in early psychosis services (Purcell et al., 2012; Purcell, Harrigan, Glozier, Amminger, & Yung, 2015), and (b) specific treatments, which in addition to targeting psychotic disorders, would also target substance use, delinquency and associated factors (Hodgins & Klein, 2017; Quinn & Kolla, 2017). Our result should additionally encourage the rapid assessment of patients' impulsivity to closely monitor their level evolution over the course of the treatment, and particularly, the evolution of impulsivity in early cannabis users. This early use could be an indicator of a possible negative evolution of impulsivity during the treatment.

### Limitations

First, the number of patients in each group was unequal and rather small to guarantee an optimal statistical power. Second, impulsivity has been assessed with two items, taken from a non-specific assessment scale. Future studies would benefit from having a more detailed evaluation of impulsivity levels to disentangle which impulsivity sub-dimensions (if any) are particularly impacted in early cannabis consumers VPs. Second, one may argue that VBs could be considered as a manifestation of impulsivity. However, to ensure they could reliably be distinguished in this study, VBs were assessed by different sources of information. Notably, the caregivers reporting on VBs were different from those assessing impulsivity. Moreover, VBs were often reported by family or friends, which was not the case of impulsivity. Furthermore, not all incidents of VB had a strong impulsive component and most could hence be planned. Third, we did not evaluated whether patients were using various doses of cannabis, which types of cannabis they used (various proportions of Tetra HC, or cannabidiol), and over which time periods, which could have had an effect on impulsivity levels. Fourth, since the diagnostic procedure focused primarily on the psychotic dimensions, it did not provide reliable information regarding the presence of a comorbid personality disorder with psychotic disorders. Finally, we must indicate the absence of pre-registered analysis plan regarding this article, which could be a limit.

## Conclusion

Our study highlighted the particular evolution of impulsivity in early cannabis users VPs. To date, no study has evaluated the effectiveness of treatment on the evolution of impulsivity over time. Further work should deepen the evaluation of impulsivity, of its different forms, and its evolution over time during treatment, particularly in conjunction with possible alterations at the neurobiological level. Clinically, our findings encourage VB risk assessment and early intervention on factors such as CU and impulsivity, which are likely to be related to these behaviors. The precocity of CU can also act as a red flag or at least a point of attention for case managers and mental health professionals involved in the treatment.

## Data Availability

The data that support the findings of this study are not openly available.
